# Migrations of the Ovum

**Published:** 1882-06

**Authors:** 


### MIGRATIONS OF THE OVUM.

To settle the question whether or not it is possible for ova to travel across part of the peritoneal cavity or that of the uterus, Dr. Leopold, of Leipsic, has performed some important experiments. In these he made use of eight rabbits. In each case he opened the abdomen, tied the right Fallopian tube in two places and cut out the piece between the ligatures ; the left ovary was carefully removed, then the abdominal wound was closed. After thorough recovery each animal was put to the male. In six cases the result was entirely negative, but in two pregnancy followed. The abdomen of the latter was opened ; in one four placentæ were found in the left horn of the uterus, and one in the right. In the other, there were three placentæ in the left horn, and two in the right. He thinks that these experiments settle the question. In these rabbits ova could only reach the uterus by travelling across the peritoneum from the right ovary to the left Fallopian tube ; and could only get into the right horn of the uterus by passing down the left horn and up the right. They prove, therefore, that it is possible for ova to migrate, not only across the peritoneum, but across the uterine cavity.—*Med. Times and Gazette.*

